# Supplementary material for: Content-rich biological network constructed by mining PubMed abstracts
Source: BMC Bioinformatics. 2004 Oct 8;5:147. doi: 10.1186/1471-2105-5-147 (PMC528731; doi:10.1186/1471-2105-5-147)
Supplement: Additional File 5 — The original Chilibot query results of the term "long-term potentiation (LTP)" and 22 other terms, limiting the latest references analyzed to the years 1990, 1995, 2000, and 2004. [file 1471-2105-5-147-S5.bz2 › chilibotAdditionalFile5/ltp2004/html/LTP_NMDA.html]

 


 **LTP** and **NMDA** 
  
Found 1302 abstracts in PubMed,  **30 abstracts were retrieved and analyzed**.  


---

 Search Google  |
 PDF files only 
|  EDU domain only 

---

**Interactive relationship** (e.g. stimulation, inhibition, etc)

- This form of  **LTP**  requires activation of  **NMDA**  receptors and D1 like dopamine receptors.  Ref: 14724247 J Neurosci, 2004
- Forskolin induced  **LTP**  in the CA1 hippocampal region is  **NMDA**  receptor dependent.  Ref: 14702333 J NeurophysiolJ Neurophysiol, 2004
- Supplement of D serine, an agonist for the glycine binding site of N methyl D aspartate  **NMDA**  receptors, enhanced  **NMDA**  receptor activation and enabled  **LTP**  induction in glial conditioned medium cultures.  Ref: 14638938 Proc Natl Acad Sci U S A, 2003
- Intracellular domains of  **NMDA**  receptor subtypes are determinants for long term potentiation  [ **LTP** ]  induction.  Ref: 14645471 J Neurosci, 2003
- Our general conclusion is that the induction of  **LTP**  caused by elevating cAMP requires presynaptic activity and  **NMDA**  channel opening.  Ref: 14702333 J NeurophysiolJ Neurophysiol, 2003
- Moreover, the N methyl D aspartate  **NMDA**  receptor inhibitor AP 5, which blocked  **LTP** , prevented the increase in the 26S proteasome activity.  Ref: 14695480 Sheng Li Xue Bao, 2003
- Long term potentiation  [ **LTP** ]   **LTP**  is generated in anoxic or hypoglycemic conditions via activation of  **NMDA**  while inhibition of these receptors atenuates this response.  Ref: 14754508 J Cell Mol MedJ Cell Mol Med, 2003
- This effect was blocked and long term potentiation  [ **LTP** ]   **LTP**  was induced by co application of N methyl D aspartate  **NMDA**  during ACPD perfusion ACPD  **NMDA**  induced LTD.  Ref: 14746918 Brain Res, 2004
- The results suggest that  **NMDA**  receptors contribute to the transient increase in 26S proteasome activity during induction of  **LTP**  in the hippocampal CA1 region.  Ref: 14695480 Sheng Li Xue Bao, 2003
- **NMDA**  receptor GluRepsilon1 KO mice showed increase in thresholds for hippocampal long term potentiation  [ **LTP** ]  and hippocampus dependent contextual learning.  Ref: 14607261 Life Sci, 2003
- RhoA and RhoB were activated by low frequency stimulation, while the induction of long term potentiation  [ **LTP** ]   **LTP**  by high frequency stimulation was associated with specific activation of RhoB via  **NMDA**  receptor stimulation.  Ref: 14622110 J Neurochem, 2003
- Surprisingly, the induction of associative  **LTP**  at cortical inputs was completely independent of postsynaptic activity, including depolarization, postsynaptic  **NMDA**  receptor activation or an increase in postsynaptic calcium concentration, and did not require network activity.  Ref: 14685239 Nature, 2003
- Using dendritic and somatic whole cell recordings, combined with calcium imaging in rat hippocampal slices, we found that the induction of long term potentiation  [ **LTP** ]   **LTP**  was accompanied by a local increase in dendritic excitability that was dependent on the activation of  **NMDA**  receptors.  Ref: 14730307 Nat Neurosci, 2004
- Previous reports using this test on the hippocampus suggest that  **NMDA**  receptor mediated mechanisms are involved in memory processing and are also necessary for the induction of long term potentiation  [ **LTP** ]   **LTP**  of the hippocampal dentate gyrus.  Ref: 14706556 PeptidesPeptides, 2004
- Co application of an inositol, 5 trisphosphate IP3 receptor inhibitor, 2 aminotheoxydiphenyl borate, during ACPD application had no effect on the ACPD  **NMDA**  induced  **LTP** , but increased the magnitude of the ACPD induced LTD, suggesting that the ACPD  **NMDA**  induced  **LTP**  involves  **NMDA**  receptors, but not IP3 receptors.  Ref: 14746918 Brain Res, 2004
- The N methyl D aspartate  **NMDA**  ion channel plays a role in neuroprotection, neurodegeneration, long term potentiation  [ **LTP** ] , memory, and cognition.  Ref: 14698791 Nucl Med Biol, 2003
- Group I mGluR LTD DP was inhibited by prior preconditioning brief high frequency stimulation HFS if the preconditioning HFS induced long term potentiation  [ **LTP** ]   **LTP**  or if the induction of  **LTP**  was inhibited by an  **NMDA**  receptor antagonist.  Ref: 14698757 Neuroscience, 2004
- In addition, the  **NMDA**  antagonist CPP blocked the occurrence of  **LTP**  at each age, and doubled the percentage of animals expressing LTD alone for all ages combined.  Ref: 14645375 J NeurophysiolJ Neurophysiol, 2004
- The induction of  **LTP**  in both mixed cultures and hippocampal slices was suppressed by  **NMDA**  receptor antagonists, glycine binding site blockers of  **NMDA**  receptors, or an enzyme that degrades endogenous D serine.  Ref: 14638938 Proc Natl Acad Sci U S A, 2003

**Parallel relationship** (e.g. studied together, co-existance, homology, etc.)

- In addition, the induction of  **LTP**  was not affected by the N methyl D aspartate  **NMDA**  receptor antagonist 2 amino 5 phosphonovalerate, suggesting that activation of  **NMDA**  receptors is not required.  Ref: 14600401 Biol Pharm Bull, 2003
- **NMDA**  receptor dependent  **LTP**  appears to be necessary for classical conditioning in Aplysia.  Ref: 14624850 Trends Neurosci, 2003
- This study examined the effects of monoamine oxidase MAO inhibitors on N methyl D aspartic acid  **NMDA**  induced perturbation of neurotransmission and normal  **NMDA**  receptor dependent function long term potentiation  [ **LTP** ] ,  **LTP**  in the CA1 field of hippocampus.  Ref: 14628188 J Neural Transm, 2003
- Simultaneous activation of converging cortical and thalamic afferents specifically induced associative, N methyl D aspartate  **NMDA**  receptor dependent  **LTP**  at cortical... inputs.  Ref: 14685239 Nature, 2003
- These results indicate that the state of  **NMDA**  receptor activation during ACPD perfusion determines whether  **LTP**  or LTD is induced in hippocampal CA1 neurons.  Ref: 14746918 Brain Res, 2004
- We present data describing the extracellular stimulation requirements for  **LTP**  induction and its long lasting maintenance >4 h at the Schaffer collateral CA1 synapse, and show that such changes in synaptic efficiency are  **NMDA**  receptor dependent.  Ref: 14583401 J Neurosci Methods, 2003
- Thus, besides  **NMDA**  receptor mediated calcium influx, subtype specific signaling is critical for  **LTP**  induction, with the intracellular C terminal domain of the NR2 subunits directing signaling pathways with an age dependent preference.  Ref: 14645471 J Neurosci, 2003
- Increased susceptibility to  **LTP**  generation and changes in  **NMDA**  NR1 and NR2B subunits mRNA expression in rat hippocampus after MCH administration.  Ref: 14706556 PeptidesPeptides, 2003
- Here we investigated the role of these  **NMDA**  receptor subtypes in long term potentiation  [ **LTP** ]   **LTP**  induction.  Ref: 14645471 J Neurosci, 2003
- They not only share common learning rules and induction pathways with the better known synaptic plasticity such as  **NMDA**  receptor dependent  **LTP**  and LTD, but also contribute in synergy with these synaptic changes to the formation of a coherent engram.  Ref: 14657257 Learn MemLearn Mem, 2003
- Selegiline reduces N methyl D aspartic acid induced perturbation of neurotransmission but it leaves  **NMDA**  receptor dependent long term potentiation  [ **LTP** ]  intact in the hippocampus.  Ref: 14628188 J Neural Transm, 2003
- According to this mechanism, an activation of postsynaptic receptors, bound to Gi 0 proteins, should increase the magnitude of  **NMDA**  dependent  **NMDA**  independent LTD  **LTP**  of excitatory inputs and  **LTP**  LTD of inhibitory inputs to all types of striatal cells.  Ref: 14658303 Usp Fiziol NaukUsp Fiziol Nauk, 2003
